# Urology. Treatise on Strictures of the Urethra, and Their Rational Treatment

**Published:** 1846-07

**Authors:** 


Art. X.—*Urologie. Traité des Angusties ou Rétrécissements de l'Urètre, et de leur traitement rationnel.* Par le Dr. Leroy-d'Etiolles.

*Urology. Treatise on Strictures of the Urethra and their rational treatment.* By Dr. Leroy-d'Etiolles.—*Paris*, 1846. 8vo, pp. 88.

Under the above fanciful title is ushered into the world a work by a man who is no doubt well acquainted with the subject of which he treats. It is one of a class written not because the author had anything new to say, but because he conceived that particular circumstances rendered it advisable that the work should be published. In 1838, the Marquis d'Argenteuil left by will to the Académie Royale de Médecine 30,000 fr., "to be placed with the interest that it will produce from the day of my death in the French Rentes, and of which the accumulated revenue shall be given every six years to the author of the most important improvement introduced during that time into the curative treatment of stricture of the urethra. In case this part of the art of cure shall not have been the object of any improvement sufficiently notable to merit the prize which I have instituted, the Academy may award it to the author of the most important improvement, during the same period, in the treatment of other diseases of the urinary organs." The Academy required that all papers should be delivered by the 21st of September 1844, and declared the value of the prize to be upwards of 8,000 fr.

Twenty-seven memoirs were received, among which we conclude was that of M. Leroy-d'Etiolles. On the 25th September a commission was chosen to examine and report. It was composed of MM. Jobert, Amussat, Villeneuve, Bérard, Lagneau, Begin, Ségalas, Civiale, Jourdan. On the 17th of December the Academy decided that the number of competitors was too great to admit of the adjudication of the prize at that public sitting, and that it would be adjudged in the course of 1845, when its value will be 10,430 fr. 50 cent.

Such, it would seem, is the circumstance to which we owe the work now before us.

We have not found in it anything new or important which would, in our judgment, entitle it to the munificent bequest of M. d'Argenteuil. And we have to complain of the mode in which everything is dilated: for instance, we have 45 pages, closely printed, on the nature of stricture; 27 pages on the seat and causes; 45 pages of diagnosis; and 250 pages of treatment; and by way of make-weight, if the stricture part should fail, 35 pages on vesico-vaginal fistulæ: besides all this, we have interminable cases of barons, deputies, diplomates, princes, councillors, lords, poets, counts, &c., some occupying five or six pages, and in number amounting to near 100; and what to us is still more unpleasant, constant squabbles with M. Civiale, M. Velpeau, &c. This is not the plan on which a good practical work should be written; though we presume that is what M. Leroy-d'Etiolles desired to do—and what, in our conscience, we believe him quite capable of doing. He has had much experience; is a man of good sense, and generally expresses himself well; and we think his book contains most things that need be said on the subject; but these things are mixed up with so much that was not wanted, that they are not easy to find. Sir B. Brodie required only 72 smaller pages to say all that he thought necessary on the subject.

There is one point on which we are less satisfied with our author,—we refer to his views on the curability of stricture;—of cured cases he gives us upwards of 50, and as he does not employ any other means than those in common use, we wonder that the result of his treatment is so different from that of men of most experience among ourselves.

The observations on vesico-vaginal fistulæ, as well as the instruments for carrying them out, are ingenious enough. With reference to these cases it is fully clear that the apparently diverse results are owing to a want of accuracy in description; some are cases in which the fissure extends little beyond the urethra; those are very manageable; in others it extends along a great part of the septum; these are very difficult of cure.

